# Knowledge, practice, counseling confidence, and intention to use AAR model of smoking cessation among respiratory therapists: A cross-sectional study

**DOI:** 10.1097/MD.0000000000035816

**Published:** 2023-10-27

**Authors:** Saleh S. Algarni, Mohammed M. Alqahtani, Fares D. Alanazi, Abdulaziz A. Alruqayti, Ibrahem S. Aghanem, Khalid A. Alajimi, Abdulhamed D. Alnkhali, Raed A. Alshahri, Turki Alhadlaq, Arwa Alruwaili, Noora Mumenah, Rayan A. Siraj, Khalid S. Alwadeai, Sami H. Alossaimi, Abdullah A. Alqarni, Ayedh D. Alahmari, Tareq F. Alotaibi, Hassan Aljohani, Taha Ismaeil, Abdullah M. Alanazi

**Affiliations:** a Department of Respiratory Therapy, College of Applied Medical Sciences, King Saud Bin Abdulaziz University for Health Sciences, Riyadh, Saudi Arabia; b King Abdullah International Medical Research Center, Riyadh, Saudi Arabia; c Respiratory Services, King Abdulaziz Medical City, Ministry of National Guard – Health Affairs, Riyadh, Saudi Arabia; d Department of Respiratory Care, College of Applied Medical Sciences, King Faisal University, Al-Hasa, Saudi Arabia; e Rehabilitation Sciences Department, College of Applied Medical Sciences, King Saud University, Riyadh, Saudi Arabia; f Department of Respiratory Therapy, Faculty of Medical Rehabilitation Sciences, King Abdulaziz University, Jeddah, Saudi Arabia; g Department of Respiratory Therapy, Batterjee Medical College, Jeddah, Saudi Arabia.

**Keywords:** confidence, respiratory therapist, smoking cessation, tobacco skills, tobacco smoking

## Abstract

There is a paucity of research on knowledge, practice, counseling confidence, and intention to use ask, advice, and refer (AAR) model of smoking cessation among respiratory therapists (RTs). Thus, we aimed to analyze the characteristics and factors that may influence them. We collected data using online questionnaires from convenience sample of active licensed RTs in Saudi Arabia. We included 206 participants. A descriptive analysis of the demographic information and characteristics of smoking cessation counseling practices and confidence were conducted. Multiple linear regression was used to test whether demographic variables and AAR model components significantly predicted the RTs’ calculated cumulative score of tobacco counseling confidence skills. Our results showed a deficiency in tobacco knowledge among RTs. Most RTs did not have certifications or attend lectures or seminars related to tobacco treatment. RTs were unfamiliar with the smoking cessation program contact information and mobile smoking cessation clinics but reported a high tobacco counseling confidence score. Clinical experience (*P* = .008), familiarity with smoking cessation program contact information (*P* = .02), inquiry regarding smoking status (*P* < .001), and advice regarding smoking status (*P* = .03) significantly predicted tobacco counseling confidence levels in RTs. RT experience, knowledge, and awareness of smoking cessation programs could enhance the confidence level among them in implementing AAR model.

## 1. Introduction

Smoking is an addictive behavior that may contribute to mental and physical attachment from nicotine dependence.^[1]^ It is linked to a wide range of fatal pulmonary and cardiovascular diseases.^[2]^ Despite the documented negative health impact of smoking, numerous people are engaged in tobacco consumption. Approximately 1.3 billion individuals smoke tobacco globally.^[3]^ Furthermore, considerable amount of research has shown that smoking prevalence is increasing among adults in Saudi Arabia. A study reported that approximately 14.09% of adults are active smokers.^[4]^ Another study conducted in Saudi Arabia demonstrated that 12.2% of adults are current smokers and majority of them (74.1%) smoked 15 cigarettes daily.^[5]^

Healthcare providers, including respiratory therapists (RTs), are considered as one of the main pillars for diagnosing, monitoring, and treating individuals suffering from cardiac, pulmonary, and other tobacco related-diseases.^[6]^ Healthcare workers play a crucial role as role models and advisors in helping people quit smoking.^[7]^ They play a pivotal role in advising and promoting smoking cessation and implementing the ask–advise–refer (AAR) model, which is devised to assess the smoking status of smokers and ultimately assist them throughout the quitting process.^[8]^

The advice from healthcare providers such as physicians was effective in increasing the quitting rate.^[9]^ Health educators, addiction counselors, RTs, and other medical experts have made significant efforts to raise public awareness regarding the risks associated with tobacco use.^[10]^ In contrast, in Riyadh, the capital city of Saudi Arabia, most physicians indicated that they rarely question their patients’ smoking habits or provide smoking cessation advice.^[11]^

Currently, only a limited number of papers have discussed the role of RTs in smoking cessation.^[10,12]^ The studies conducted in North America found that the percentage of RTs trained in counseling is limited; both studies were limited by high percentage of female RTs, i.e., 88% and 100%. To the best of our knowledge no other study was conducted among RTs in other countries except for North America.

RTs are responsible for managing many chronic lung diseases that are mostly caused by tobacco smoking. Therefore, the role of RTs in advising patients during the management is key for recovery and good rehabilitation. Therefore, we aimed to evaluate knowledge, practice, counseling confidence, and intention to use AAR model of smoking cessation among RTs in Saudi Arabia. This study extends the existing knowledge regarding integrating RTs in smoking cessation program as key healthcare providers in smoking cessation.

## 2. Methods

This quantitative cross-sectional study was conducted from July 2021 to December 2021. RTs working at different hospitals in Saudi Arabia were invited to participate in this study as they were the main target population. Inclusion in the study was contingent on participants being from Saudi Arabia and working as RTs at hospitals in Saudi Arabia. The questionnaire was distributed anonymously among RTs through social media channels such as WhatsApp, Twitter, and Telegram. A convenience sampling technique is used. No sample size calculation was performed, as this was an exploratory study. All respondents were included in the study.

The participants were notified of the study’s purpose, and their consent was obtained before they filled out the questionnaire. Ethics approval was obtained from the King Abdullah International Medical Research Center before conducting this study (IRB SP21R/229/05).

### 2.1. Measure

A valid and reliable questionnaire was adapted from a previous study.^[12]^ The questionnaire contained 28 items comprising multiple choices, yes-no, and Likert scale. Questions were constructed to obtain information on demographic characteristics (sex, geographical region, educational attainment, experience, working area, and smoking status) and assess practice and knowledge related to smoking cessation services (being a certified tobacco treatment specialist, attended smoking treatment or counseling lectures/seminars, knew the phone number of smoking cessation programs, and learnt about mobile smoking cessation clinics).^[12]^

To evaluate counseling skill confidence, participants were asked to rate their current practice, intention to implement the AAR approach, and confidence in 13 items on a scale of 0 to 10, wherein 0, 5, and 10 indicated not confident, moderately confident, and extremely confident, respectively. Additionally, smoking cessation counseling skill confidence was adopted from the same source.^[12]^ The final score for smoking cessation counseling skill confidence was computed as the mean of each subscale for the participants (1 = low and 10 = high). A higher mean score indicated greater confidence in smoking cessation counseling skills among the participants. Noteworthy, the internal consistency for the questionnaire was estimated by Cronbach alpha coefficient (0.907 for the overall) and a range of 0.897 to 0.914 for all included domains.

### 2.2. Statistical analyses

Descriptive analyses were conducted to improve our understanding of participants’ characteristics. Multiple linear regression was conducted to examine the associations between the intention to use the AAR model (dependent variable) and the following predictors: confidence in smoking cessation counseling skills, demographic information, practice, and knowledge of smoking cessation service use. SPSS version 26 was used for all the analyses. Statistical significance was set at *P* < .05.

## 3. Results

### 3.1. Participants characteristics

Of the 206 participants, most were male, lived in the central region of Saudi Arabia, and had a bachelor’s degrees. Most of the RTs who participated in this study indicated that they had <3 years of experience working in intensive care units and were not currently smoking. Most RTs who participated in this study did not hold tobacco treatment certifications or attend lectures or seminars related to tobacco treatment. Moreover, most RTs were unfamiliar with the smoking cessation program contact information and mobile smoking cessation clinics (see Table 1).

**Table 1:** Sociodemographic characteristics of the study participants.

Variable	Frequency N (%)
*Sex* Female Male	80 (38.8) 126 (61.2)
*Geographical region* Central region Eastern region Western region Southern region Northern region	117 (56.8) 47 (22.8) 30 (14.6) 7 (3.4) 5 (2.4)
*Educational attainment* Associate degree Bachelor’s degree postgraduate degree	7 (3.4) 183 (88.8) 16 (7.8)
*Respiratory care clinical experience* 0–3 years 3–5 years +5 years	121 (58.7) 30 (14.6) 55 (26.7)
*Working area* Outside intensive care units Intensive care units	48 (23.3) 158 (76.7)
*Smoking status* Not current smoker Current smoker	148 (71.8) 58 (28.2)
*Tobacco treatment specialist Certification and Licensure* No Yes	191 (92.7) 15 (7.3)
*Attendance of smoking treatment or counseling lectures/seminars* No Yes	145 (70.4) 61 (29.6)
*Knowledge about the phone number of smoking cessation program* No Yes	158 (76.7) 48 (23.3)
*Knowledge about mobile smoking cessation clinics* No Yes	138 (67.0) 68 (33.0)

### 3.2. Participants’ use of AAR model

We analyzed implementation of the AAR technique by RTs in patients who smoked. Most RTs asked the patients whether they smoked in the previous week and indicated that they did not advise quitting to patients who smoked or referred them to a smoking cessation counseling session or the national tobacco control program (see Table 2).

**Table 2:** Descriptive of use of and intention to use the AAR model among respiratory therapists in Saudi Arabia.

Use of AAR model In the past week, did you…	
	Percentage N (%)
*Ask a patient whether he/she smokes?* No Yes	86 (41.7) 120 (58.3)
*Advise a patient to quit smoking?* No Yes	114 (55.3) 92 (44.7)
*Refer a patient to smoking cessation counseling?* No Yes	174 (84.5) 32 (15.5)
*Refer a patient to the national smoking cessation program?* No Yes	186 (90.3) 20 (9.7)
*Intention to use AAR model* *Please describe your intention to routinely implement the following smoking cessation activities…*	
*Asking all (or almost all) patients whether they smoke?* Not considering doing this Considering doing in the next few months Considering doing in the next few weeks Already do	56 (27.6) 25 (12.3) 33 (16.3) 89 (43.8)
*Advising all (or almost all) currently smoking patients to quit?* Not considering doing this Considering doing in the next few months Considering doing in the next few weeks Already do	62 (30.4) 34 (16.7) 45 (22.1) 63 (30.9)
*Discussing and providing currently smoking patients with materials for calling the national smoking cessation Quitline (937*)? Not considering doing this Considering doing in the next few months Considering doing in the next few weeks Already do	81 (39.9) 40 (19.7) 55 (27.1) 27 (13.3)

AAR = ask–advise–refer.

### 3.3. Intention to use AAR model

We analyzed the intention to routinely implement the AAR technique. Most RTs reported having already asked patients whether they smoked. Moreover, the participants had nearly equal intention to not advise and advise all (almost or nearly all) currently smoking patients to quit (see Table 2).

### 3.4. Participants’ counseling skill confidence and self-efficacy

Table 3 shows the self-reported rate of participants’ confidence on a scale of 0 to 10, in which 0, 5, and 10 represented not confident, moderately confident, and extremely confident, respectively. The calculated cumulative score of tobacco counseling skill confidence and self-efficacy among RTs is nearly more than half of the total confidence score; the mean cumulative score is 82.8, which is about 63.7% confidence level; this percentage was obtained by dividing our mean score for the whole group by the total score of counseling skill confidence and self-efficacy, which was 130 level (see Table 3).

**Table 3:** Level of confidence in smoking cessation counseling skills among respiratory therapists in Saudi Arabia.

Variable	N	Mean (SD)
How confident are you that you…		
Can routinely ask patients whether they use tobacco?	203	7.3 (2.8)
Can sensitively suggest tobacco cessation to patients who use tobacco?	203	6.9 (2.6)
Know the appropriate questions to ask patients when providing tobacco cessation counseling?	203	6.0 (2.6)
Can provide/enhance motivation among patients who want to quit?	203	6.6 (2.6)
Have sufficient knowledge to discuss smoking cessation medications and appropriate use?	203	5.4 (2.8)
Know when a referral to a physician is appropriate?	203	6.1 (2.6)
Are able to provide adequate counseling when time is limited?	202	5.6 (2.5)
Can help patients learn how to cope with situations or triggers that might lead them to relapse to using tobacco?	203	6.0 (2.5)
Can encourage patients who are not ready to quit to think about quitting in the near future?	202	6.5 (2.5)
Can discuss smoking cessation with pregnant women?	202	6.9 (2.6)
Can counsel the parents of pediatric patients to quit smoking?	203	6.4 (2.7)
Can include a family member in a discussion about tobacco cessation?	202	6.4 (2.5)
Can counsel adolescents about tobacco use?	202	6.4 (2.5)
Cumulative score	198	82.8 (27.1)

SD = standard deviation.

### 3.5. Multiple regression analysis

Multiple linear regression was used to test whether the demographic variables and AAR model components significantly predicted the RT’s calculated cumulative score of tobacco counseling skill confidence, and self-efficacy. We have tested the linear regression assumptions, including normality, linearity, independence, homogeneity, and multicollinearity. We found that our data met all the assumptions successfully. It was found that male sex and having a higher degree were significant predictors of high tobacco confidence levels. Furthermore, having 3 to 5 years of experience significantly predicted lower tobacco confidence levels than having more than 5 years of experience. Additionally, familiarity with smoking cessation program contact information significantly predicted low tobacco confidence levels. However, the intention to ask all (or almost all) patients whether they smoked and having already asked patients whether they smoked significantly predicted high tobacco confidence levels. Similarly, intention to advise all (or almost all) currently smoking patients to quit significantly predicted high tobacco confidence levels (see Table 4).

**Table 4:** Multivariable linear regression model for predicting smoking cessation counseling skill confidence among respiratory therapists in Saudi Arabia.

Independent variables	Smoking cessation counseling skill confidence		
	β	t	*P* value
*Sex* Female Male	Ref 0.16	Ref 2.09	Ref .03*
*Educational attainment* Associate degree Bachelor’s degree Postgraduate degree	Ref 0.20 0.24	Ref 1.78 2.04	Ref .07 .04*
*Experience* 0–3 years 3–5 years +5 years	Ref −0.20 −0.10	Ref −2.69 −1.38	Ref .008* .17
*Working area* Outside intensive care unit Intensive care unit	Ref −0.02	Ref −0.32	Ref .75
*Smoking status* Not current smoker Current smoker	Ref −0.04	Ref −0.49	Ref .62
*Certified tobacco treatment specialist* No Yes	Ref 0.08	Ref 1.18	Ref .24
*Attendance of smoking treatment or counseling lectures/seminars*. No Yes	Ref 0.03	Ref 0.49	Ref .63
*Knowledge of the contact information of a smoking cessation program* No Yes	Ref −0.18	Ref −2.29	Ref .02*
*Knowledge about mobile smoking cessation clinics* No Yes	Ref 0.13	Ref 1.77	Ref .08
*In the past week, did you ask a patient whether he/she smokes* No Yes	Ref −0.07	Ref −0.96	Ref .34
*In the past week, did you advise a patient to quit smoking* No Yes	Ref −0.01	Ref −0.17	Ref .86
*In the past week, did you refer a patient to smoking cessation counseling* No Yes	Ref 0.04	Ref 0.41	Ref .68
*In the past week, did you refer a patient to the national smoking cessation program* No Yes	Ref −0.01	Ref −0.15	Ref .88
*Intention to asking all (or almost all) patients whether they smoke* Not considering doing this Considering doing in the next few months Considering doing in the next few weeks Already do	Ref 0.09 0.22 0.36	Ref 1.03 2.33 3.36	Ref .30 .02* <.001*
*Intention to advising all (or almost all) currently smoking patients to quit* Not considering doing this Considering doing in the next few months Considering doing in the next few weeks Already do	Ref 0.06 0.19 0.22	Ref 0.58 1.85 2.11	Ref .56 .06 .03*
*Intention to discussing and providing currently smoking patients with materials for calling the national smoking cessation Quitline (937*) Not considering doing this (Reference group) Considering doing in the next few months Considering doing in the next few weeks Already do	Ref 0.05 0.09 0.11	Ref 0.57 0.95 1.21	Ref .57 .34 .22

* *P* ≤ .05; Ref = reference group.

## 4. Discussion

The current study sheds light on knowledge, practice, counseling confidence, and intention to use AAR model of smoking cessation among RTs in Saudi Arabia. This study found that most RTs are unfamiliar with the smoking cessation program and do not recommend or refer smokers to it. Importantly, the RTs’ confidence in implementing AAR approach is hindered by their knowledge, experience, and awareness.

This study is essential because healthcare providers who help in quitting smoking have proven to be effective in combating tobacco-related problems. Furthermore, increasing the amount of behavioral support provided by healthcare providers is likely to increase the chances of success.^[9]^ Our findings reflect some characteristics of RTs in terms of providing smoking cessation services.

Comparison of the findings with those of 2 previous studies^[10,12]^ confirms that the smoking cessation counseling practices of RTs are still limited as observed by the percentage of RT involvement. This may support the hypothesis that RTs are not actively involved in smoking cessation practice.

Notably, most RTs did not attend any tobacco training lectures or seminars, a low percentage of RTs had tobacco treatment credentials, and very few were unfamiliar with smoking cessation program contact information or mobile smoking cessation clinics. This can be explained by that smoking cessation domain is a minor part of RTs’ education and training.^[13]^

The presented study could not demonstrate a link between attending educational lectures or seminars in improving confidence in conducting counseling sessions. This outcome is contrary to a previous study that showed that RTs who were trained during or after their studies had significantly better counseling practices for both patients who were ready to quit and those who were not than RTs who did not receive any training.^[10]^ This can be explained by the extensive training as presented in the previous study as an intervention where our study is considers educational activities. This spurred concern over the importance of implementing extensive tobacco training among RTs.

Our findings revealed that most RTs reported high confidence levels in advising and referring smokers to counseling sessions. The confidence level of counseling skills plays a vital role in the current practice of implementing the AAR technique because improving confidence among healthcare providers would enhance their ability to implement the AAR technique like before.^[14]^

This study has clinical implications and provides valuable information to clinicians and decision-makers regarding the importance of integrating tobacco cessation training in RTs training programs. This will facilitate tobacco cessation counseling and subsequently decrease the number of smokers that RTs receive. Further work should be undertaken in future to investigate the presences of smoking cessation modules in RTs’ educational curriculum.

The current study has several limitations. First, we used a self-reported questionnaire, which may increase the potential for response bias. Second, we used a cross-sectional design which provides only a snapshot of what occurs during data collection and allows conclusions about associations but not causality. The temporal relationship between the independent and dependent variables cannot be definitively established. Additionally, this study was limited by exploratory design that aimed to investigate RTs’ practice regarding smoking cessation. Moreover, majority of respondents are male, and this might intervene with the conclusion of this study. Finally, utilizing a closed-format questionnaire could prevent the collection of additional data that might have been obtained in an open-ended questionnaire. The participants in this study were from hospitals in Saudi Arabia. Thus, the study findings should be cautiously generalized to all RTs.

## 5. Conclusions

The confidence of RTs in the implementation of smoking cessation counseling could be spurred by their experience, knowledge, awareness of the smoking cessation program, and their intention of asking and advising patients regarding their smoking habits. Continuous efforts are required to promote extensive training and education in enhancing smoking cessation counseling practices among RTs.

## Author contributions

**Conceptualization:** Saleh Algarni, Abdullah M. Alanazi.

**Formal analysis:** Ayedh Alahmari, Abdullah M. Alanazi.

**Investigation:** Fares D. Alanazi, Abdulaziz A. Alruqayti, Ibrahem S. Aghanem, Khalid A. Alajimi, Abdulhamed D. Alnkhali, Raed A. Alshahri, Turki Alhadlaq, Arwa Alruwaili, Noora Mumenah, Sami H. Alossaimi, Tareq Alotaibi.

**Methodology:** Saleh Algarni, Rayan A. Siraj, Abdullah A. Alqarni, Tareq Alotaibi, Hassan Aljohani.

**Project administration:** Saleh Algarni.

**Software:** Arwa Alruwaili, Noora Mumenah.

**Writing – original draft:** Rayan A. Siraj, Khalid S Alwadeai, Abdullah A Alqarni, Taha Ismaeil.

**Writing – review & editing:** Saleh Algarni, Mohammed M. Alqahtani, Abdullah M. Alanazi.
